# The flowering of Atlantic Forest *Pleroma* trees

**DOI:** 10.1038/s41598-021-99304-x

**Published:** 2021-10-14

**Authors:** Fabien H. Wagner

**Affiliations:** Geoprocessing Division, Foundation for Science, Technology and Space Applications – FUNCATE, São José dos Campos, SP 12210-131 Brazil

**Keywords:** Biogeography, Tropical ecology, Image processing, Forest ecology, Macroecology, Flowering, Computational science

## Abstract

Mapping the spatial distribution of a plant is a current challenge in ecology. Here, a convolutional neural network (CNN) and 33,798 Sentinel-2 satellite images were used to detect and map forest stands dominated by trees of the genus *Pleroma* by their magenta-to-deep-purple blossoms in the entire Brazilian Atlantic Forest domain, from June 2016 to July 2020. The *Pleroma* genus, known for its pioneer behaviour, was detected in an area representing 10.8% of the Atlantic Forest, associated negatively with temperature and positively with elevation, slope, tree cover and precipitation. The detection of another genus by the model, 18% of all the detections contained only pink blooming *Handroanthus* trees, highlighted that botanical identification from space must be taken with caution, particularly outside the known distribution range of the species. The *Pleroma* blossom seasonality occurred over a period of ~5–6 months centered on the March equinox and populations with distinct blossom timings were found. Our results indicate that in the Atlantic Forest, the remaining natural forest is less diverse than expected but is at least recovering from degradation. Our study suggests a method to produce ecological-domain scale maps of tree genera and species based on their blossoms that could be used for tree studies and biodiversity assessments.

## Introduction

**Figure 1:**
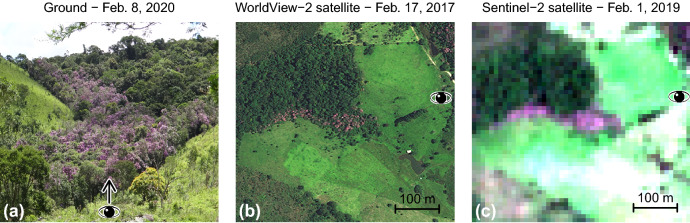
Same scene of a blooming *Pleroma pulchra*-dominated forest taken from different points of view: from the ground (**a**), from the satellite WorldView-2 at a very high spatial resolution of 50 cm (**b**) and from the satellite Sentinel-2 at a spatial resolution of 10 m (**b**). The center of the scene is located at latitude − 23.27502°S and longitude − 45.18446°W. These *Pleroma* trees grew on a pasture that was abandoned after 1962, while the other parts of the forest were already present in 1962. A complete map of the *Pleroma* in the WorldView-2 images and a history of the tree cover in this region is presented in^6^. WorldView-2 satellite image courtesy of the DigitalGlobeFoundation.

For about two centuries, the Atlantic Forest of Brazil, well known as a biodiversity hotspot and global priority for conservation^1,2^, has undergone an intense deforestation and degradation^3^. Today, less than 15% of its original area remains, distributed throughout extremely fragmented forest patches^4,5^. As a consequence, some pioneer species, such as tree species of the genus *Pleroma*, have taken advantage of these degraded conditions and are now extremely common in the Atlantic Forest landscapes^6^. The *Pleroma* genus (formerly assigned to the genus *Tibouchina*) belongs to the Melastomataceae family and is only present in the Neotropics^7^. It was first described by the botanist Jean Baptiste Christophore Fusée-Aublet in 1775 in French Guiana near an abandoned house^8^. Remarkably, his text also first described *Pleroma* trees pioneer and dominance characteristics: he highlighted their local abundance in the newly colonized space. The *Pleroma* genus currently contains ~240 known species that typically have magenta or deep purple flowers and are particularly well represented in eastern Brazil—where are found ~30% of all species of this genus^9,10^. In the Atlantic Forest, species of *Pleroma* are commonly known as *Manacá da serra* and *Quaresmeira*, the latter meaning literally ’tree of Lent’ because its blossoms coincide with Lent (mid-February—early April). *Manacá da serra* refers mainly to the species *Pleroma pulchra* and *Pleroma mutabilis*, while *Quaresmeira* refers mainly to *Pleroma granulosa*. In the remnants of Atlantic Forest, *Pleroma* trees grow mainly in clusters where they are abundant or dominant, on forest borders or on abandoned land. The synchronous and exuberant magenta or deep purple blossoms they exhibit for several weeks during the first months of the year, make them visible even from long distances (Fig. 1a). Due to these characteristics, it is not surprising that a blooming *Pleroma*, *Pleroma granulosa Cogn.*, was among the first collected plants (sampled during collection n°1) by the botanist Carl Friedrich Philipp von Martius in Rio de Janeiro during his 1817–1820 expedition to Brazil with the zoologist Johann Baptist von Spix. *Pleroma granulosa Cogn.* was later described in the book collection *Flora Brasiliensis* among 22,767 other plant species collected during this expedition^11,12^. Today, *Pleroma* trees are arguably some of the most well-known and popular Atlantic Forest trees.

The characteristics of abundance and synchronous flowering render this genus of great interest to studies of plant spatial distribution and flowering timing^6,13,14^. While it should be acknowledged that in a forest that harbours ~20,000 plant species^1^, other tree species with flowers of similar colours certainly exist—such as species from the genus *Handroanthus*, popularly know as *Ipê* in Portuguese or *Lapacho* in Spanish—there are no other tree species besides *Pleroma* with magenta or deep purple flowers that make such large dominant to monospecific clumped forest stand, which is why they are easily recognisable in satellite imagery (Fig. 1b,c). Specifically, in the Sentinel-2 images, *Pleroma* can be identified with high confidence because their monodominant clumped stands appeared as continuous patches of magenta/purple color (Fig. 1c), where individual tree crowns are not visible, and that can exhibit various shapes and sizes. At the difference of the trees of similar color, such as *Handroanthus* trees, that can dominate the forest canopy with crown larger than 10 m of diameter, *Pleroma* trees have a relatively small stature, with 8-12 m height, and their blooming is highly visible in the 10 m of spatial resolution of Sentinel-2 only when several blooming individuals are clumped together in dense stand, a common behaviour of this pioneer species. Consequently, we also have to acknowledge that the absence of visible blooming in the Sentinel-2 image does not guarantee that there is no *Pleroma* tree in the image, as isolated *Pleroma* blooming trees are just not visible at this 10 m spatial resolution.

In the last 10 years, significant efforts have been made to map blooming of canopy-emergent tropical tree species in remote sensing images, mainly at Barro Colorado Island (BCI)—Panama. For example, more than 700 flowering *Handroanthus guayacan* were mapped over the island using high resolution images (2.4 m) acquired in the same season (April–March) of two different years^15^. Later in BCI, three emergent trees species with conspicuous flowers, *Handroanthus guayacan*, *Dipteryx panamensis* and *Jacaranda copaia*, and several inconspicuous species were mapped in very high-resolution aerial image (0.129 m) to test if species crown distributions alone could be used to estimate species distribution and spatial autocorrelation observed from the field^16^. In 2015, the blooming of *Handroanthus guayacan*, *Dipteryx panamensis* and *Jacaranda copaia*, were also used to identify the species from satellite images and then the authors demonstrated that it was possible to identify these species using imaging spectroscopy based solely on the optical reflectance properties of non-flowering tree crowns with accuracies above 94%^17^. Furthermore, always at BCI, with a map of 1,006 *Handroanthus guayacan*, the number unique detection (2,596) and of observation attempts (18,883) during an 11-yr period, it was demonstrated possible to remotely estimate adult mortality rates for a canopy tree species precisely^18^. This dataset of *Handroanthus guayacan* was later used to test whether adult recruitment of *H. guayacan* was negatively density dependent^19^. In the Peruvian and Colombian Amazon, thousands of flowering individuals of yellow and pink crowns with synchronous flowering, that could be potentially for the studies of population dynamics, have been already observed in high-resolution images from the Planet Labs constellation of cube-sats and remained to be mapped^20^.

Highly conspicuous blooming *Pleroma* trees have already been mapped successfully in satellite images of very high spatial resolution (50 cm × 50 cm) in a the region overlapping the largest Atlantic Forest remnant near São Paulo^6^. This work was the first to map a blooming tree species at a regional scale and ~4757 ha of blooming *Pleroma* dominated forests were mapped, likely representing several thousands, if not millions, of individuals. However, even if very high resolution images are currently the best images to map individuals or groups of the same tree species, they have two main limitations: they are not freely available and no consistent time series exists to study plant phenology. One currently available option for obtaining high-resolution images to map the *Pleroma* trees are the images provided by the Sentinel-2 satellites. With two satellites, launched in June 2015 and in March 2017, the Copernicus Sentinel-2 mission currently provides, among other products, high-resolution images with 10 m of spatial resolution and four spectral bands—red, green, blue and NIR—distributed in tiles covering 10,000 km^2^^21^. The frequency of revisit is of five days at the Equator and enables to monitor Earth’s surface changes and plant phenology^22,23^. Furthermore, in these 10 m spatial resolution images, blooming *Pleroma* forest patches are visible, their colours rendering them detectable and separable from the forest and other landcover (Fig. 1c).

To map or detect trees in these Sentinel-2 satellite images, the tools available currently that hold great promise belong to deep learning methods and are known as convolutional neural networks (CNNs)^24^. These CNNs have been already used successfully to map tropical tree species by their crown particular shapes and leaf colors, such as the species *Bertholletia excelsa* (Brazil nut trees) and *Cecropia hololeuca*^25,26^; and also by their blossoms, such as the species *Pleroma Pulchra* in the Atlantic forest^6^. Recently They were also used for the task of tree species recognition in temperate forests^27^. Even though they were developed recently, CNNs have already been applied to a large variety of problems relating to the remote sensing of vegetation; furthermore, as has been observed since 2012 in the computer vision field^28^, CNNs systematically outperform traditional (or shallow) machine learning methods^29^. For the particular case of tropical tree species identification in the Atlantic forest domain, it was shown that traditional machine learning methods were faster and could, to a certain extent, reach similar accuracies to those attained by the CNNs, however they were less stable to changes in datasets and highly dependent on prior segmentation and hand-engineered features^30^. The principal advantage of using CNNs in remote sensing is their accuracy, which is similar to human-level classification and detection accuracy—but is consistent and fast—enabling rapid application over very large areas and/or through time^31,32^.

**Figure 2:**
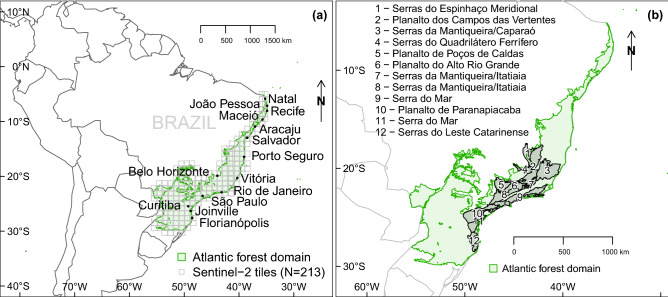
Geographical location of the Atlantic Forest domain in green, extents of the 213 Sentinel-2 tiles in light grey and main cities within the domain (**a**). Geographical locations and local names of principal mountain chains and high plateaus of the Atlantic Forest domain, geomorphological units respectively named *Serras* and *Planalto* in Portuguese^33^ (**b**).

For the purpose of this investigation, mapping blossoming *Pleroma* trees, two options were available: semantic segmentation, in which each pixel is classified individually (such as U-net model)^26,32,34^ or detection, which only classifies the image as containing the object or not (such as the VGG16 model)^35^. The later was chosen. The detection of *Pleroma* trees were made in images of 1.28 km of side, obtained by splitting the Sentinel-2 tiles by 80 rows and 80 columns. A total of 33,798 selected Sentinel-2 images belonging to 213 tiles covering the Atlantic Forest were used for the detection on the period from June 2016 to July 2020. The Sentinel-2 tile locations, main cities, and main mountains and high elevation plateaus of the domain are presented in Fig. 2a,b. Detection was chosen over segmentation for two main reasons. First, it enabled to manually produce a training sample relatively fast (< 1 day for one Sentinel-2 image), while segmenting more than one Sentinel-2 image by hand would probably take weeks or months. Second, detection is simpler and less computationally intensive, drastically reducing the time of processing and subsequent analysis. Given the scale of the Atlantic Forest, it was also considered sufficient to make a presence-absence map of *Pleroma* trees at 1.28 km spatial resolution for each Sentinel-2 image rather than a map pinpointing exact locations of the trees. The timing of *Pleroma* flowering within each areas of 1.28 km × 1.28 km where *Pleroma* trees have been detected can be determined simply by observing the frequency of blooming detections per month.

Such large-scale species maps could contribute, for example, to understand species distribution, to locate species of interest for conservation, or to understand components of population dynamics, such as flowering, recruitment or mortality.

In this work is presented (i) a map showing *Pleroma* trees presence in the entire Atlantic Forest, as detected by their magenta or dark purple blossoms using Sentinel-2 images and a detection deep learning algorithm, (ii) their flowering cycles and (iii) the relationship of *Pleroma* trees’ spatial distribution to environmental and climatic characteristics.

## Results

### Blossom presence and detection frequency

**Figure 3:**
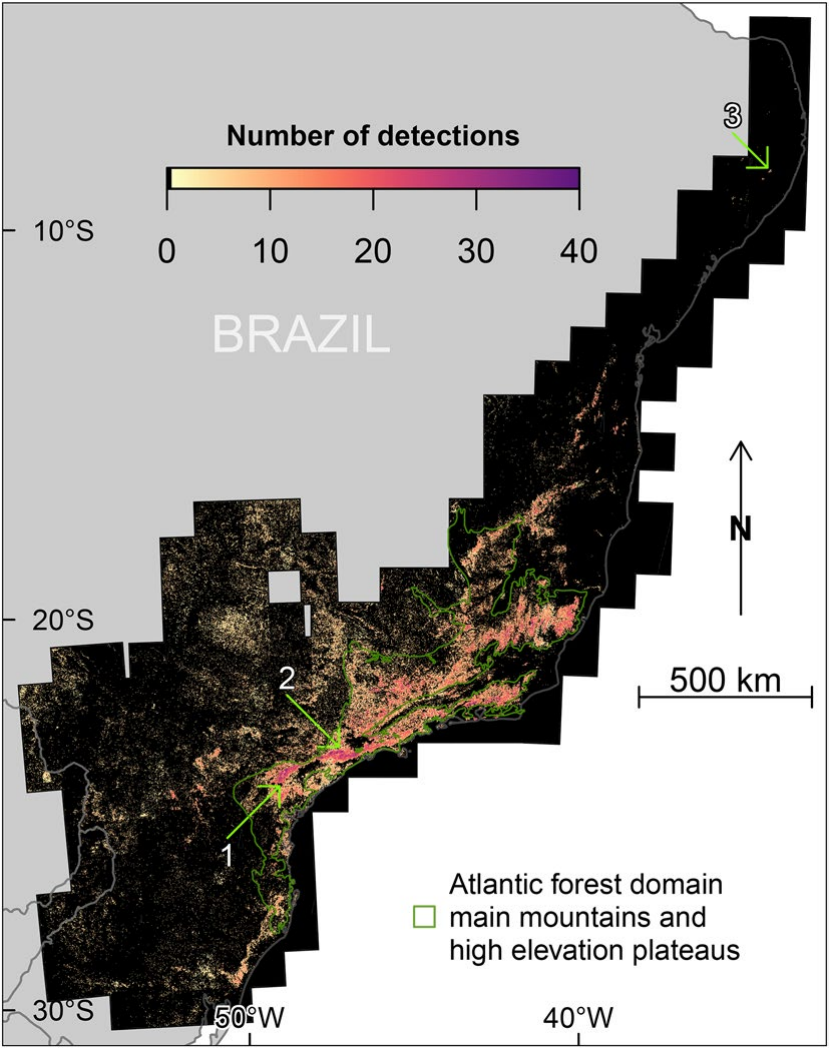
Pink and magenta blossoms detections in the Atlantic Forest domain in percentage of cloud free images (%) during the 4-year period between June 2016 and July 2020. Each pixel is a square of 1.28 km × 1.28 km. A detection indicates that the model has found pink-magenta blooming trees in the Sentinel-2 image over this area. Pixels with less than 4 detections during the study period were set to zero. Arrows 1 and 2 indicate regions in the north of the city of Curitiba and in the south of the São Paulo city, respectively. Arrows 3 indicates the northern detected forests with pink and magenta blooming trees.

**Figure 4:**
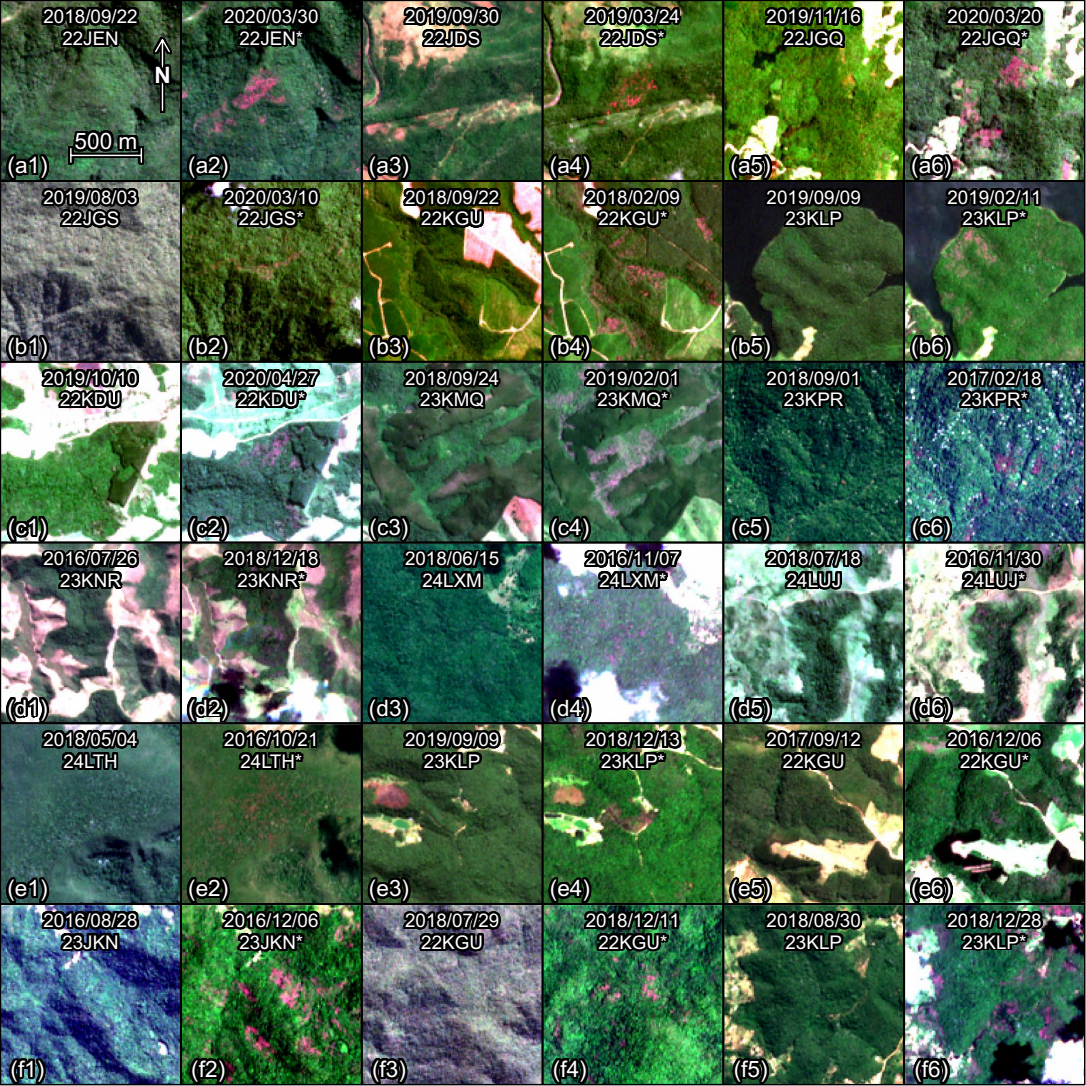
Sentinel-2 image subsets with *Pleroma* trees detected by the model (Panel I). To ease the visual detection of the pink/magenta trees, one image taken outside the blossom period and one taken within the blossom period (denoted with an *) are given for each region of detection. The dates and tile names of the Sentinel-2 images are indicated on each.

**Figure 5:**
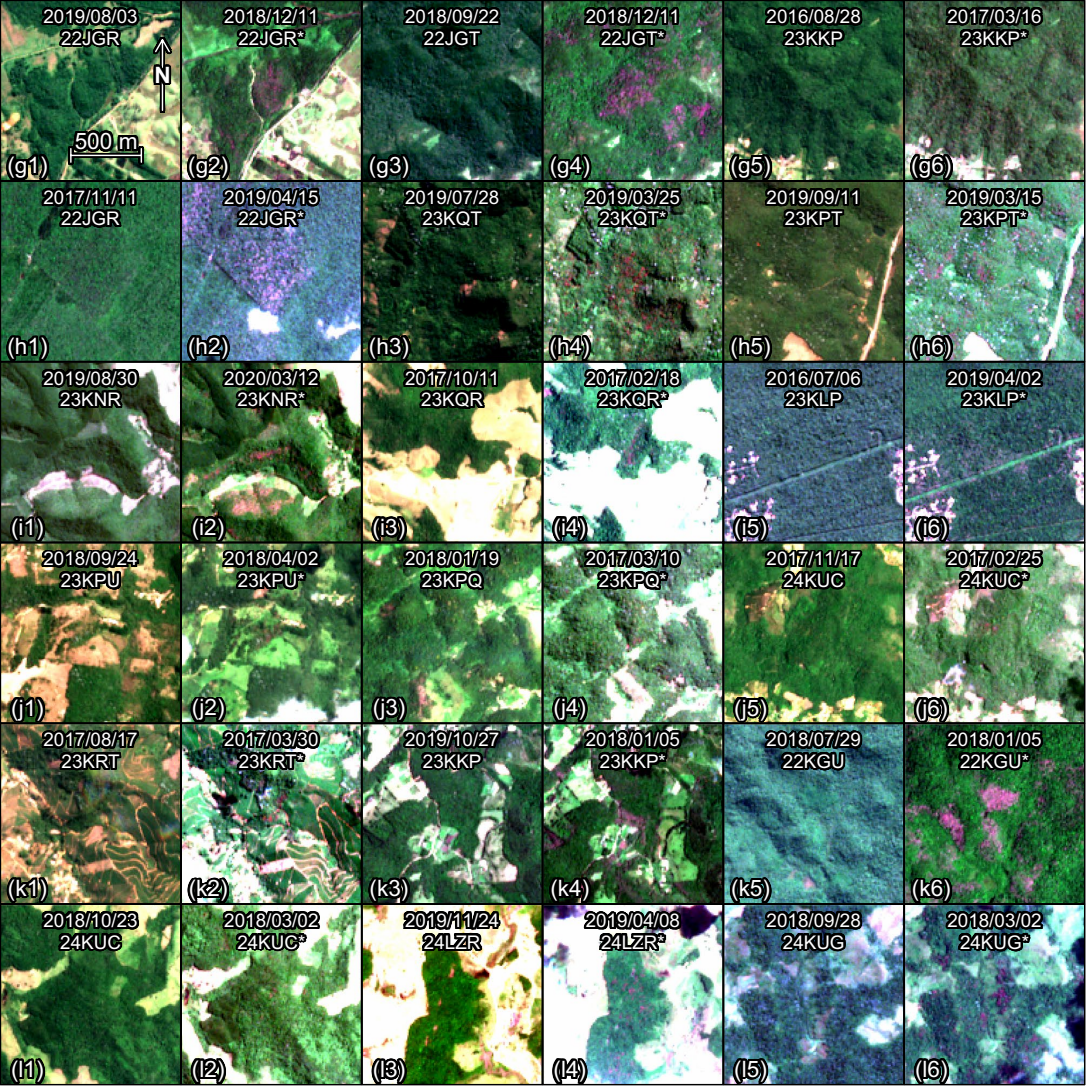
Sentinel-2 image subsets with *Pleroma* trees detected by the model (Panel II). To ease the visual detection of the pink/magenta trees, one image taken outside the blossom period and one taken within the blossom period (denoted with an *) are given for each region of detection. The dates and tile names of the Sentinel-2 images are indicated on each.

**Figure 6:**
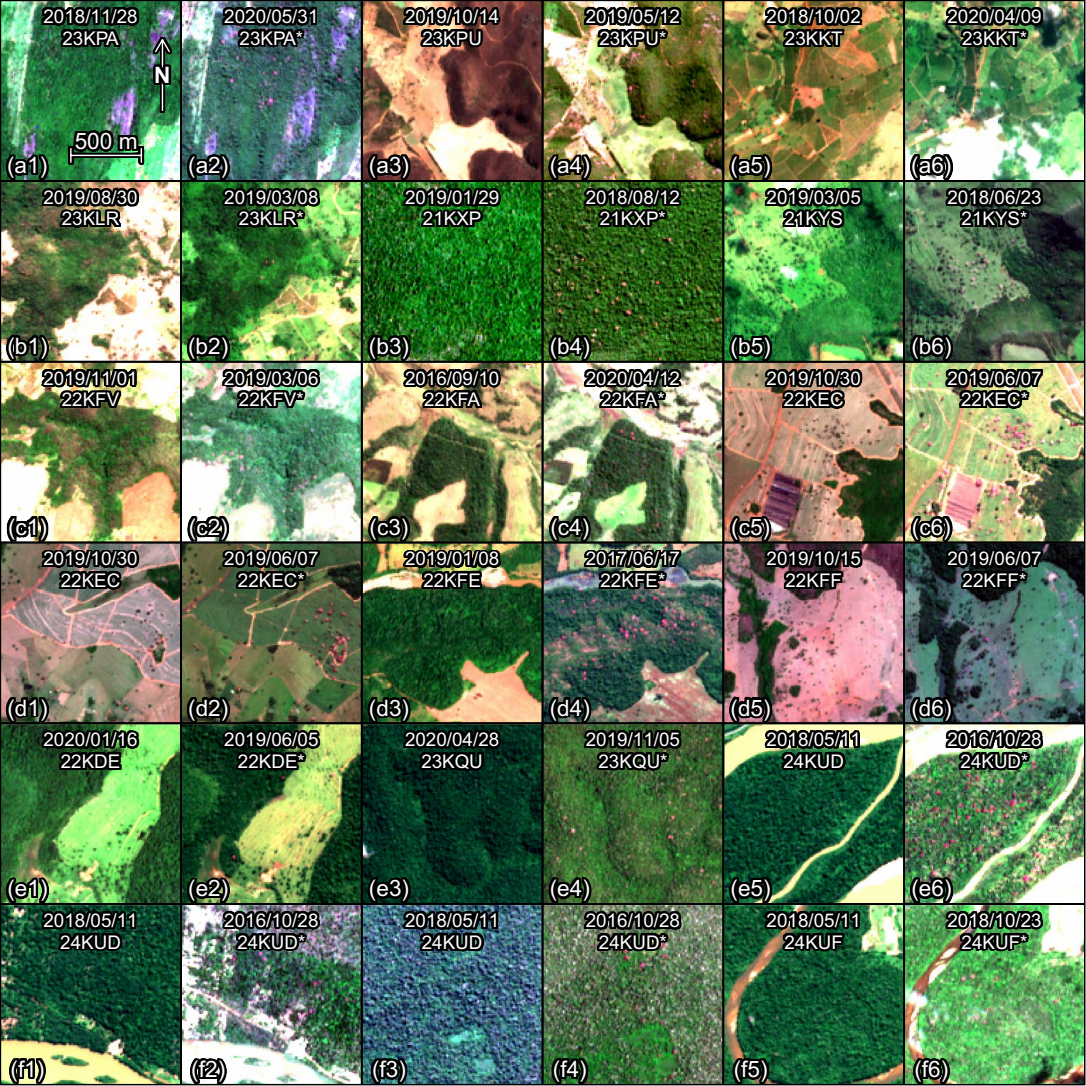
Sentinel-2 image subsets with *Handroanthus* trees detected by the model (Panel III). To ease the visual detection of the pink/magenta trees, one image taken outside the blossom period and one taken within the blossom period (denoted with an *) are given for each region of detection. The dates and tile names of the Sentinel-2 images are indicated on each.

The detection model was applied to the 33,798 Sentinel-2 images during the 4-year period between June 2016 and July 2020 to detect the presence of blooming *Pleroma* trees and to compute the frequency of detection per area of 1.28 km × 1.28 km over the Atlantic Forest (Fig. 3). Trees with pink or magenta blooms, consisting primarily of *Pleroma* trees, and in a lesser proportion of large *Handroanthus* trees, that had at least one detection per year were found in 13.2% of the Brazilian Atlantic Forest pixels (139,960 pixels) (Fig. 3). The median number of detections of blooming trees was 6.5% of the cloud-free images (95% confidence interval—from percentile 2.75 to 97.5—of 2.6 to 23.3%). Spatial patterns appeared when observing the distribution and the percentage of detections in cloud-free images. At the biological domain scale, the observed pattern of detection seems related to the increasing and generally high elevations on the Atlantic coast side (see elevation and slope in Fig. 12a,b). These geographical units are mountain chains known as the *Serras* (Fig. 2b), and 26.6% of the detections were made in the five following mountain chains : Serra do Mar (9.3%), Serra da Mantiqueira (Caparaó: 7.5% and Itatiaia: 6.6%), Serras do Leste Catarinense (1.2%), Serras do Quadrilátero Ferrífero (1.1%) and Serras do Espinhaço Meridional (1%) (Figs. 3 and 2b). Furthermore, 17.9% of the detections were made in high elevation plateaus (known as *Planalto* in Portuguese) in the vicinity of the previous Serras, such as the Planalto de Paranapiacaba (6.2%), Planalto dos Campos das Vertentes (5.7%), Planalto do Alto Rio Grande (3%) and Planalto de Poços de Caldas (3%) (Figs. 3 and 2b). A total of 44.5% of all detections were made in these geomorphological units, forming a continuous area coinciding with the detection area, a region delimited in green in Fig. 3. At a regional scale, spatial correlations of detection rates are observed, which is relevant considering that the detections are made per pixel. Several spatially consistent regions of high detection density (>20% of the cloud-free images) that were larger than 100 km were observed, for example, in the north of the city of Curitiba (arrow 1, Fig. 3) and in the south of the São Paulo city (arrow 2, Fig. 3), which are located in the the region formed by the Planalto de Paranapiacaba plateau and the Serra do Mar mountains. Several examples of images with blossoms detected, as well as the same images outside the blossom period, are given in Figs. 4, 5 and 6. Within the Atlantic Forest, the northernmost region with detected blooming trees was in the State of Pernambuco, in hills located 7 km southeast of the city of Bonito (arrow 3, Fig. 3). However, even these latter trees shown similar color and dominant behaviour as *Pleroma*, we must remain cautious because the presence *Pleroma* was unexpected in this region.

The typical appearance of *Pleroma* trees detected by the model is magenta clusters of pixels, mostly without visible crowns (Figs. 4 and 5). The sizes of the forest patches vary from some pixels of 10 × 10 m to large patches of several hectares—most being continuous and fewer being sparsely distributed. They are mainly found within or on the border of the forest. In contrast, the *Handroanthus* mostly detected in the eastern parts of the Atlantic Forest and in few areas on the Atlantic coast side (Fig. 6), present different characteristics: very large crowns (of several 10 × 10 m pixels) that can be identified individually and are found within the forest, occasionally on its border, and oftentimes in pastures. They can be found isolated or in groups with more than 10 individuals.

### Flowering seasonality

**Figure 7:**
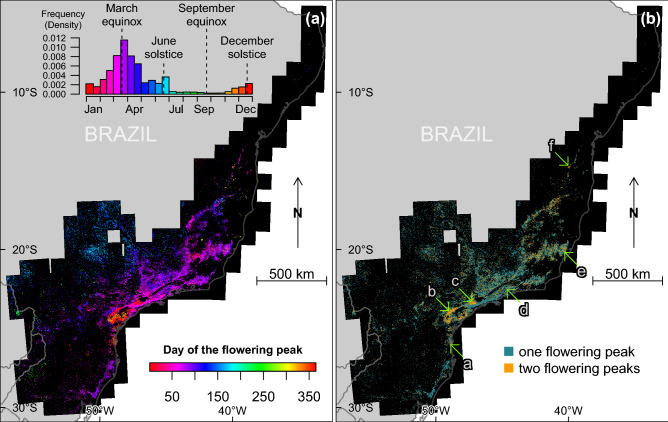
Day of the flowering peak (**a**) estimated from the mean monthly detection time series and Fourier transform signal decomposition (see Methods). For the pixel showing two flowering peaks in per year in (**a**), only the highest peak is represented. Number of flowering peaks per year (**b**). Subset images of locations indicated by arrows are given in Fig. 8. The flowering peaks on the map are mainly from trees of the genus *Pleroma* and in a lesser proportion from large trees of the genus *Handroanthus* that can be also detected.

**Figure 8:**
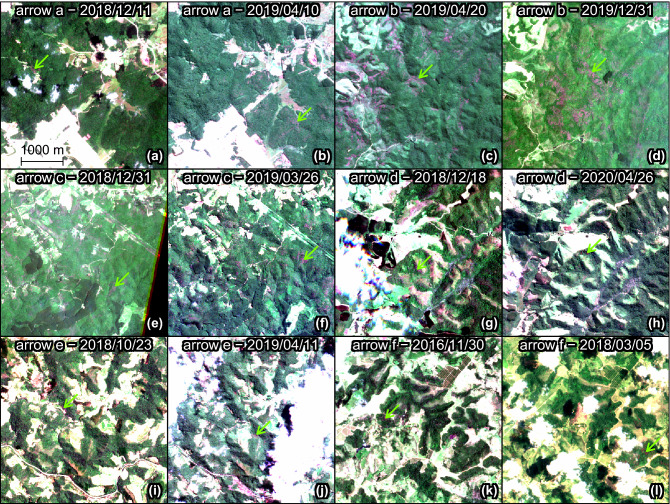
Sentinel-2 image subsets (4 km × 4 km) for the locations with two blossoms per year. Locations of the arrows are given in Fig. 7b. A green arrow shows one of the location of trees with pink flowers in each image. Image equalization with min-max stretching between 0 and 255 for each RGB band was performed to improve contrast and better enable visual detection of the pink flowering trees.

To analyse the seasonality of blooming, the time series of detections per month were computed per area of 1.28 km × 1.28 km and filtered using the Fourier transform (FT) to provide a continuous representation of the discrete blooming observations and find if there was one or more blooming peaks per year and the days of the year when the blooming begins, peaks and stops (Fig. 7). Spatial patterns also appeared when looking at the date of the flowering peaks, that is, the moment of the year when more pink or magenta-blooming trees were detected (Fig. 7a). No north-south gradient was observed but rather gradients at the regional scale, most of which were continuous (Fig. 7a). 81% of the peaks occurred between February and June included, and the highest frequency of peaks was observed in March, centered around the March equinox. Two secondary blooming peaks were observed, one occurring around the December solstice and the other around the June solstice. The latter was almost entirely seen in pixels representing area far from the Atlantic coast on the eastern side of the Atlantic Forest. During and around the September equinox, only a few isolated and clustered areas blossomed in a region between 18°–20°S and 39°–43°W and on the eastern side of the Atlantic Forest. The median duration of flowering was 80 days days (95% confidence interval—from percentile 2.75 to 97.5—of 41 to 168 days). One peak occurred in 76.2% of the pixels, two peaks in 22.8%, and more than two peaks in a marginal 1% (Fig. 7b). The pixels with two flowering peaks per year showed a significantly higher number of detections per year than the pixels with only one peak per year (median of 8.5% and 6.1% of cloud-free images, respectively; Wilcoxon rank sum test p-values <2.2e-16). In the regions with two flowering peaks, two populations of *Pleroma* existed that flowered at different times (Fig. 8a–l) and were also spatially separated (mainly visible in Fig. 8c,d). Even with a trained eye, it can be difficult to spot the pink flowering trees (for example Fig. 8i–l), because the pink or magenta forest patches are small and because some other land cover types, such as bare ground, can have a pink hue. While visually observing changes between two pictures at different times is usually necessary to find and confirm the presence of pink flowering trees, the algorithm was able detect the flowers with one image only. Also, note that in the Figs. 4, 5, 6 and 8 the image bands were adjusted by minimum-maximum stretching to make easier the visualisation and comparison of images that have different illumination conditions. This had to be done manually for visual detection of the pink trees, while the model detected the pink trees independently of the illumination conditions.

**Table 1:** Characteristics of the groups obtained by K-means clustering, using flowering dates and coordinates, and sorted by flowering peak date: ID number of the group, number and frequency (%) of pixels, date of the flowering peak, identification of the genus detected in the cluster (*Pleroma* and/or *Handroanthus*) and sample of image subsets for each cluster. Note that a pixel can belong to two clusters if it has two flowering peaks.

Cluster ID	Pixels number	Frequency (%)	Peak date	*Pleroma* presence	*Handroanthus* presence	Image samples
2	1846	1.12	03-fev.	x		Figure 5k3–k6
3	7057	4.27	10-fev.	x		Figure 5k1–k2
14	14630	8.85	08-mar.	x		Figure 4b3–c4
15	10316	6.24	13-mar.	x		Figure 4a1–b2
7	17804	10.77	18-mar.	x		Figure 5h3–i4
8	20222	12.23	22-mar.	x	x	Figures 5g5–h2, 6c1–c4
5	24687	14.93	23-mar.	x	x	Figures 5i5–j4, 6a5–b2
13	2168	1.31	27-mar.	x		Figure 4c5–c6
1	13695	8.28	06-apr.	x	x	Figures 5l1–l6, 6a1–a4
4	7123	4.31	15-apr.	x		Figure 5j5–j6
11	23036	13.93	02-jun.		x	Figure 6d1–e2
6	6821	4.13	07-jul.		x	Figure 6b3–b6
12	5919	3.58	24-nov.	x	x	Figures 4d1–e2, 6e3–f6
10	2030	1.23	06-dec.	x		Figure 4e3–e6
9	7968	4.82	07-dec.	x		Figures 4f1–f6, 5g1–g4

**Figure 9:**
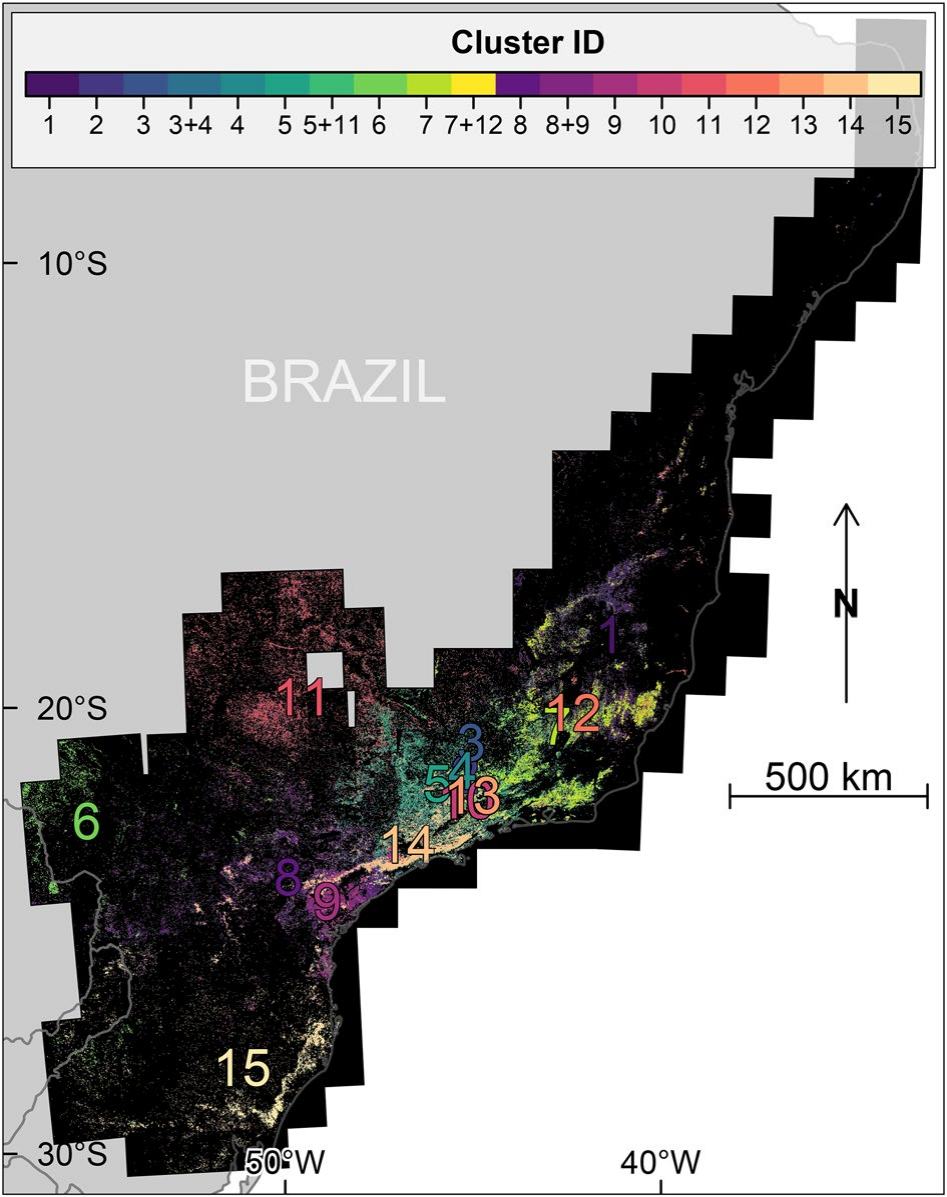
Map of the 15 clusters obtained by K-means clustering of flowering dates (start, peak and end) and detection coordinates. A pixel can belong to two clusters if it has two flowering peaks. The more frequent combinations, represented by more than 1000 pixels, are also given (3 and 4 for example). The cluster ID numbers are located at the centroid of each cluster.

*Pleroma* trees were detected by the model and identified visually in 13 of the 15 clusters obtained by K-means clustering of flowering dates (start, peak and end) and detection coordinates (Table 1 and Fig. 9), representing 10.8% of the Brazilian Atlantic Forest pixels and 82.6% of all detected blooming pixels. Among these thirty clusters (Fig. 9), seven were spatially well defined and presented in relatively uniform regions (15, 8, 9, 14, 5, 7 and 1 in Fig. 9), and six (3, 4, 2, 10, 13 and 12 in Fig. 9) presented in smaller spatial aggregations of pixels (for example, cluster 10). Most of these latter clusters had centroids at a nearby location (Fig. 9) because they all covered an area ranging from southern cluster 9 and northern cluster 1. The majority of the pixels in these 13 clusters were located on the mountains of the Atlantic coast side and were present to a lesser extent inland, as can also be observed by the position of their centroids (Fig. 9). The first clusters to bloom were clusters 12, 9 and 10 at the end of November and early December (Table 1); their main patches were located between the Atlantic coast and the cities of Joinville-Curitiba-São Paulo and also in all the Serra da Mantiqueira mountains northeast of Rio de Janeiro. In early February, clusters 2 and 3 started blooming. They are slightly more aggregated near the Curitiba-São Paulo axis that in the rest of the Atlantic Forest. In early March, cluster 14 peaks: this cluster was made up of the typical *Pleroma* of the Serra do Mar (Fig. 1)^6^. Surprisingly, its primary southern expansion did not follow the Atlantic coast but rather the mountain chain located on the São Paulo-Curitiba axis. Five more clusters had a flowering peak after cluster 14 during March (clusters 15, 7, 8, 5, 13 in Fig. 9). Cluster 15 was the southernmost and most homogeneous *Pleroma* cluster. Going North, cluster 8 overlapped spatially with cluster 9 and 14. Cluster 5 extended from the São Paulo-Rio de Janeiro axis to the state of Minas Gerais. Cluster 13 was small but had a relatively large distribution. Cluster 7 was the dominant cluster in the mountain chains northeast of Rio de Janeiro. *Pleroma* clusters 1 and 4 experienced peak blooming in April. Cluster 4 overlapped with cluster 3 in more than 1000 pixels and 4 was also distributed in all regions of *Pleroma* clusters. The last cluster to peak is cluster 1, which ranges from the north-northeast Vitoria and extends north, and where were found the northernmost *Pleroma*, near the city of Bonito, Pernambuco (arrow 3 in Figs. 3 and 5l3–l4). All the *Pleroma* blooming peaks were concentrated in only five months: from December to April.

In the two clusters where no *Pleroma* were identified visually, 11 and 6, the model detected isolated trees of the genus *Handroanthus* with large pink blooming crowns (2.8% of the Brazilian Atlantic Forest pixels, Table 1). Both *Pleroma* and *Handroanthus* were identified visually only in four other clusters: cluster 1 (the northernmost cluster), cluster 12, and clusters 5 and 8, which both overlapped with cluster 11 (Fig. 9 and Table 1). In the region of overlap, more than 1000 pixels displayed two flowering peaks in a combination of clusters 5 and 11 (Fig. 9), corresponding to a first peak of *Pleroma* trees at the end of March and a second peak of *Handroanthus* trees in early June (Table 1). Clusters 11 and 6 were spatially adjacent with peaks only one month apart.

**Figure 10:**
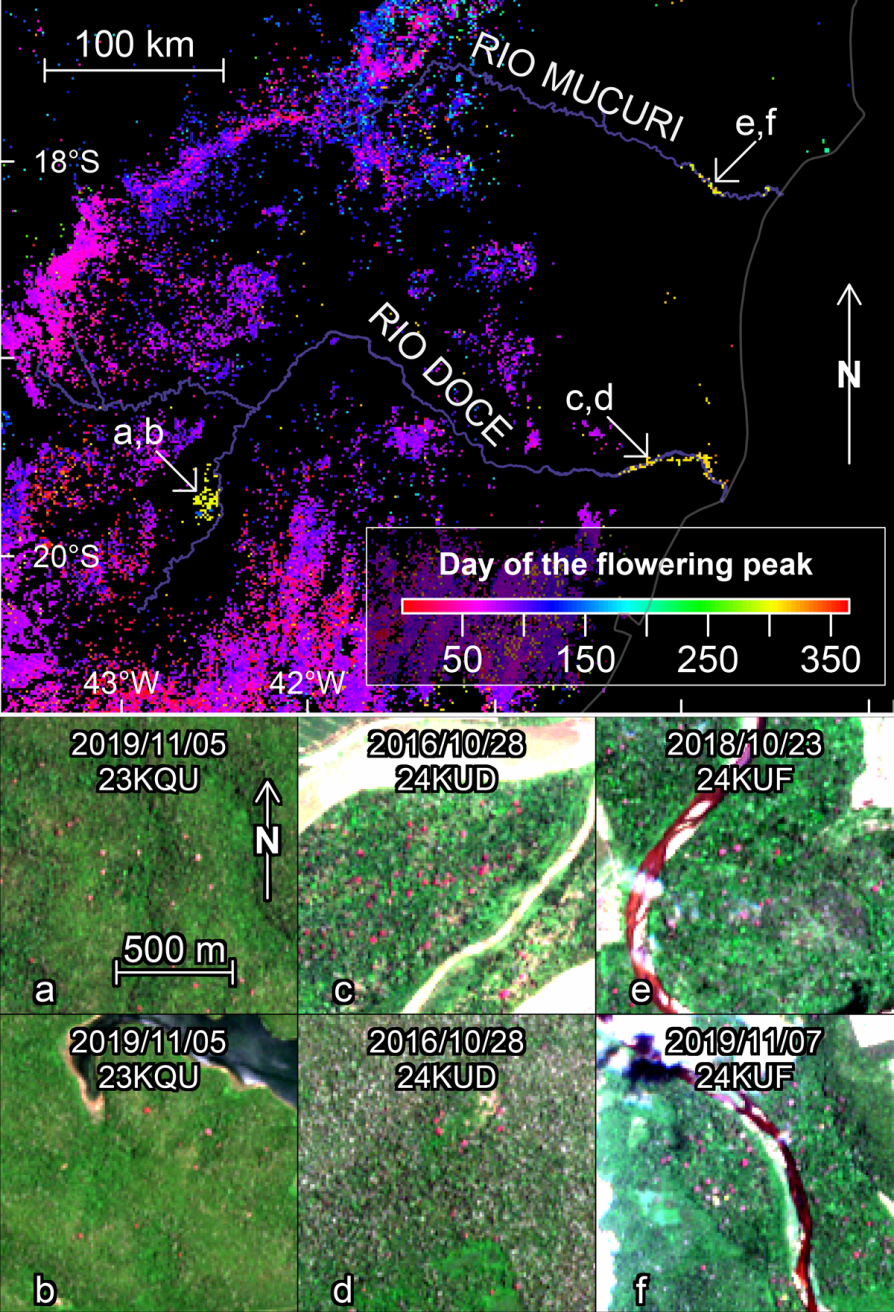
Geographical locations of the three isolated patches with trees of the genus *Handroanthus* flowering in late October/early November (from cluster 12) and Sentinel-2 image subsets of locations of the pink flowering trees in Rio Doce State Park (**a**,**b**) and along the Rio Doce (**c**,**d**) and Rio Mucuri rivers (**e**,**f**).

Three isolated patches belonging to cluster 12 with only large pink blooming *Handroanthus* that bloom each year at the same time were found in the states of Minas Gerais, Espírito Santo and Bahia (Fig. 10). One was located along the Rio Doce river near the delta, the second along the Rio Mucuri river (also near the delta, 430 km north of the Rio Doce delta) and the third in Rio Doce State Park which is crossed by the Rio Doce river (and is near its source). Rio Doce State Park, is the largest remnant of Atlantic Forest in the Minas Gerais State and also contains a large system of lagoons. Some of the detected trees had massive crowns, with some individuals appearing in up to three or four 10 m × 10m pixel Sentinel-2 pixels in width (Fig. 10), indicating crowns with diameters between 20 and 40 m.

### *Pleroma* and *Handroanthus* tree distribution associations with environmental and climate variables

**Figure 11:**
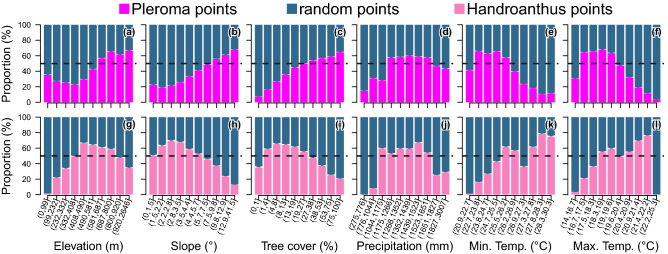
Comparison of the proportion of *Pleroma* trees (**a**–**f**) and *Handroanthus* trees (**g**–**l**) in relation to the proportion obtained by random sampling over the same area, for the quantile classes of elevation, slope, tree cover, precipitation, minimum and maximum temperatures. Barplots of the first and second row are computed respectively with 112039 observations of *Pleroma* (all clusters excluding 6 and 11) and 26177 observations of *Handroanthus* (clusters 6 and 11, located on the western part of Atlantic Forest domain). All these observations are compared to the same number of random locations over the Atlantic Forest. The random locations are bootstrapped 100 times to create the 95% confidence interval. For example, in the first row, for a given elevation class, if the confidence interval intersects with 50% (black dashed line), there is no significant difference between the distribution of *Pleroma* and a random distribution for this class at 5% significance level.

The positive association of *Pleroma* trees with increasing and higher elevations was confirmed by testing, for the variables elevation and slope, the difference between the spatial distribution of *Pleroma* (N = 112039; all clusters excluding 6 and 11) and a random spatial distribution (Fig. 11a,b). *Pleroma* trees were found in higher quantity than expected from a random spatial distribution in all elevations above ~600 m (Fig. 11a). This association was also present for slope: significantly fewer *Pleroma* are observed in slight slopes than in steeper slopes (>9.8°, Fig. 11b). *Pleroma* trees also exhibit the same pattern in relation to tree cover (Fig. 11c); they are observed in high numbers in regions with 27% or higher tree cover. In relation to climate variables, the observed distributions are consistent and highlight the *Pleroma* trees’ preference for the mountains of the Atlantic Forest (Fig. 11d–f). *Pleroma* are also observed in high numbers in areas with annual precipitation ranging from 1180 to 1650 mm per year. In terms of temperatures, *Pleroma* trees are found in high numbers in four of the five first quantiles, that is, with maximum temperatures (annual mean) ranging from 23.8°C to 26.2°C and minimum temperature (annual mean) ranging from 16.7°C to 19.6°C.

The association of *Handroanthus* trees (clusters 6 and 11) with elevation, slope, annual precipitation and temperature differs from that of *Pleroma* trees in all variables. *Handroanthus* trees are present in higher numbers in elevations between 408 and 800 m, slopes <5.7 and tree cover between 1.27 and 27.2% (Fig. 11g–i). Among climate variables, *Handroanthus* trees are found in high numbers in areas with annual precipitation ranging from 1040 to 1650 mm per year (Fig. 11j). In contrast to *Pleroma* trees, *Handroanthus* trees are found in high numbers in the hottest quantiles for maximum and minimum temperatures (Fig. 11k,l). Note that the absence of *Handroanthus* in the last quantile of minimum temperature is an artefact, as these minimal temperatures are only found on the northern part of the Atlantic coast.

## Discussion

**Figure 12:**
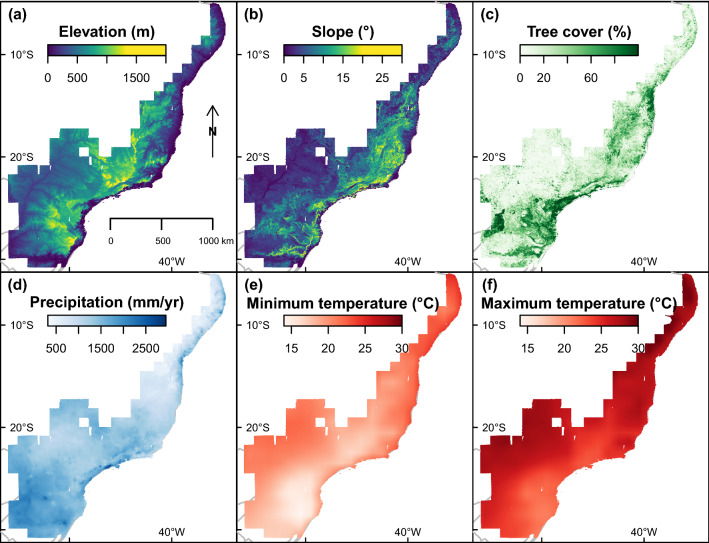
Environmental and climatic variables used in the study to analyse spatial distribution of *Pleroma* trees (**a**) elevation (m), (**b**) slope (°), (**c**) tree cover (%), (**d**) mean annual precipitation (mm yr^−1^), (**e**) annual mean of minimum temperatures (°C), and (**f**) maximal annual temperatures (°C).

This study presents a map showing the distribution in a given ecological domain of forests dominated by a tree genus based on the detection of its blossoms. Our model trained to detect blooming tree of the genus *Pleroma* detected pink and magenta blossoms in a large part of the Atlantic Forest: 10.8% (114,171 pixels) of our grid with a spatial resolution of 1.28 km × 1.28 km covering the entire domain (Fig. 3). Here, we have assumed that the probability of failing to detect forest stands of blooming *Pleroma* trees was trivially small, mainly because of the number of images available during the 4-year period of observation (~135 images with less than 80% of cloud cover for each Sentinel tiles). However, some *Pleroma* dominated forest stands could have been missed due to cloud cover or missing data and in future works, with a longer Sentinel-2 time series, we will improve the map and compute the probabilities associated to the blooming detections^18^. *Pleroma* trees were absent in ~18% of the detected blooming pixels (clusters 6 and 11, Fig. 9 and Table 1). In this region, located at more than 250 km of the Atlantic coast, where *Pleroma* trees were not expected, the detections were mainly isolated trees with large crowns of diameters >10 m and pink flowers of the genus *Handroanthus*. In the remaining ~82% of blooming detections, two patterns were identified. First, regions with only *Pleroma* (clusters 2, 3, 4, 7, 9, 10, 13, 14 and 15, Fig. 9 and Table 1), mainly located on the principal mountain chains and high plateaus of the Atlantic Forest domain that constitute the main distribution range of some well know *Pleroma* species such as *P. pulchra*, *P. mutabilis* and *P. granulosa*. Second, regions located around the main range of these species with both *Pleroma* and *Handroanthus* trees with pink flowers (clusters 1, 5, 8 and 12, Fig. 9 and Table 1). In a future work, the detection map will be confronted to known locations of conspicuous blooming species to decipher if other large canopy species than Handroanthus could have be detected. We also have to acknowledge that even the features used to detect *Pleroma* trees visually are very specific of this genus (intense magenta-to-deep-purple color, green homogeneous color outside the blooming season, dominated or continuous magenta-to-deep-purple patches and no visible individual crown), it might exist other tree species with similar features that could explain detections, in particular where *Pleroma* trees were not expected, such as the northern detected blooming trees near the city of Bonito in the Pernambuco State. The distribution of *Pleroma* trees showed a large-scale spatial pattern: they were mainly found in a large consistent patch spread over the principal mountain chains of east-southeast Atlantic Forest (Fig. 3). Furthermore, they were found preferentially in regions of high elevation, high slope and high tree cover of the Atlantic Forest (Figs. 11 and 12). *Pleroma* trees distribution association with climate also reflected their preference for the mountain environments of Atlantic Forest, and they were found in high numbers in regions with a mean annual precipitation above ~1200 mm, a mean minimum temperature of 16.7°C and a mean maximum temperature of 26.2°C. The presence of *Pleroma* trees provided indications about recent Atlantic landscape history, as they are indicators of forest regeneration and grow preferentially on abandoned pasture and the borders of forest fragments^6^. The presence of these trees indicated that previously exploited areas were abandoned in the last decades and that the current Atlantic Forest natural tree cover is composed partially of natural secondary forests with consequently less biodiversity than is typically found in a primary forest. While their distribution association with high elevation, slope and tree cover could reflect their natural range, it might also reflect that they grew on land that had been abandoned due to inaccessibility—and in the vicinity of old forests whose existence is closely linked to the same terrain characteristics (high elevation and steep slope; see Fig. 12). The federal law 5.106 of 1966, which encouraged reforestation with fiscal incentives by allowing farmers to apply 50% of their income tax to reforestation, could be responsible for this apparent increase of secondary forest cover^6,36^. For biodiversity studies and conservation, the map of detection produced by this investigation could support the production of a map of secondary forests dominated by *Pleroma* and help locate older and more diverse forest remnants.

A far more complex situation than originally expected was discovered regarding flowering cycles, with flowering peaks occurring at different times of the year, sometimes in the same area (Fig. 7), but some common characteristics of the *Pleroma* blossom could be determined. The highest frequency of peaks occurred around the March equinox, and 81% of the peaks occurred between February and June (Fig. 7a). The histogram of flowering detection peaks presents a normal distribution shape, indicating that the blossoms, even if they have different flowering times, were concentrated around a date that appeared important for the *Pleroma* genus: the March equinox (20 March), when day and night have equal duration. Homogeneous populations with similar flowering times and locations were produced with K-means clustering (Fig. 9). In these populations, we saw that, while most *Pleroma* bloomed around March, some started much earlier at the end of November to early December (clusters 9, 10 and 12; Fig. 9 and Table 1). Almost all the *Pleroma* populations obtained by clustering showed degrees of overlap: only the southern population (cluster 15) seemed to represent a spatially isolated population. It is still unknown if these flowering variations indicate different species or not, an investigation that must be undertaken through field work. In the following, we refer to these different populations as ’phenological species’. Two peaks of flowering were found in 23% of the distribution area detected by the model (Figs. 7b and 8), which indicates that this methodology is not yet sophisticated enough to individualize populations based on the temporal signal of detections, since it was evident that a mixture of phenological species occured commonly. Further research could employ detections on a grid with higher resolution; or a pixel-based segmentation—that is, determining whether each Sentinel-2 pixel contains *Pleroma* trees^6^. Sometimes, *Pleroma* trees grow in the same area, but flowering of the patches is separated temporally and spatially (for example Fig. 8c,d), and give natural experimental conditions to support biological investigations. For example, if the trees are of the same species, these conditions could help to understand what triggers flowering at different times of the year; or, if the trees are of different species, they could further our understanding of the process of colonisation and species competition. While it is too early to analyse climate effects on the blossom timing, mainly because of the mixed signal observed, some observations and assumptions can be made. First, precipitation, while important for the *Pleroma* distribution (Fig. 11), does not seems to be a direct trigger of flowering. *Pleroma* blooming was observed at different times of the rainy season (from around October to April) roughly corresponding to summer in the southern hemisphere. Second, some synchronicity at large scales (e.g. clusters present in all the main mountains of Atlantic Forest; Fig. 9) seems to indicate that flowering timing could be more linked to seasonal changes in day length and solar intensity, drivers of blossom previously suggested for tropical trees^37^ and, with addition of temperature, for Atlantic Forest trees^38^. Interestingly, while most of the observed phenological species bloomed during decreases in day length *after* 20 of December, a few phenological species (such as cluster 9) bloomed during increasing in day length *before* 20 of December, suggesting that if day length is a driver of flowering, *Pleroma* trees can sense both increases and decreases in day length. Finally, all observed signals were clearly seasonal and occurred each year during the same months, indicating that each population had a predetermined blooming time, even in populations of the same genus. Field investigations of phenology in tropical forests could use *Pleroma* trees as a model since we now know the location of an unprecedented number of individuals, as well as their flowering times. As to why *Pleroma* trees bloom during this period of the year, our assumption is that these species are adapted to colonize landslides in Atlantic Forest. *Pleroma* species are pioneer species very efficient at colonizing large disturbed spaces without forest, such as pasture^6^, further, they are found mainly on slopes and in areas with high elevations (Fig. 11a,b). In Atlantic Forest, the only natural phenomena that causes large-scale deforested disturbances are landslides. They are frequent, and, among the 699 detected in the from period 1991 to 2012 in Brazil, 80% occurred in the region of *Pleroma* distribution^39^. These landslides occur mainly during the rainy season between October and April with a peak in January^39^. Landslides could be one of the reasons *Pleroma* flourish and disperse their many seeds (>100 seeds per fruit^13^) during this period, when large spaces are opened up by these landslides, yielding land ready for colonization by *Pleroma* trees. Mapping spatial patterns of plant genera at regional scales, such as the *Pleroma* populations of Atlantic Forest, and the bamboo and palm populations in the Amazon^40,41^, is a recent field of study in tropical forests and could be used to explore broader scientific questions about tropical species distribution, flowering strategies and colonisation.

Another advantage of accessing flowering phenology is that it can help to further separate detections of phenological cycle characteristics. *Handroanthus* trees, for example, were present in ~21% of the pixels detected by the model, mainly in the western part of the Atlantic Forest domain and in some isolated populations in the eastern part (Fig. 9). The main characteristics that differ between detected *Handroanthus* trees and *Pleroma* trees is that *Handroanthus* trees individuals are visible and have very large crowns (several pixels) with pink flowers and, in the western part of Atlantic Forest, occur not only in forest fragments but also in cultivated fields (clusters 11 and 6). It has likely been one of the few tree species maintained by the farmers after clear-cutting (Fig. 6). Another interesting finding that was made with the blossom timing, are the large pink *Handroanthus* trees found in Rio Doce State Park and along the Rio Doce and Rio Mucuri rivers (Fig. 10). They present similar characteristics: synchronous flowering in late October and early November (a period when none of their direct neighbor flourish), crown sizes >25–30 m, environmental locations in the vicinity of large amounts of water (Rio Doce State Park has the third largest lake system in Brazil, and both other areas are located along both rivers). These features form a body of evidence pointing to their possibly belonging to the same species, or at least the same genus. However, the complete absence of detection between the three areas where they were found is surprising, and, unfortunately, the more likely explanation is that all the other exemplars of large pink *Handroanthus* trees in these regions have vanished, of which mining could be the culprit. The Rio Doce River, for example, has been mined for alluvial diamonds, gold and other minerals since the colonization period, after Sebastião Fernandes Tourinho found green and blue gems (likely tourmaline) along its tributaries in 1572^42^. The large *Handroanthus* trees of these three regions could have been preserved by the wet and flooded natural environment, as well as by legal protection (Rio Doce State Park has existed since 1944). The *Handroanthus* trees detected here, if confirmed as a unique species, could be used as a model species for tropical phenology with Sentinel-2. Using *Handroanthus* as a model species was also pointed out by previous works on *Handroanthus* trees^18,19^, which argued that *Handroanthus* and other tree species with conspicuous phenology are model organisms for questions in landscape-scale population dynamics because they are observable throughout large areas using remote sensing. In the case of the Atlantic forest, they have the advantage of being in regions with distance ranging from ~250–500 km that have slight climatic differences that could help disentangle the effect of climate variables. Furthermore, the model detected numerous large trees that can be easily individualized, geolocalized and followed every year with Sentinel-2 images, both to track for flowering and for leaf flush and fall.

This investigation demonstrated that detecting blossom timing of a tree genus at the ecological domain scale is now feasible with 10 m spatial resolution Sentinel-2 images (Figs. 3 and 7). Some previous attempts have already been successful at mapping tropical tree species or genera by their flowering with commercial high-resolution imagery at landscape and regional scales^6,15^, but using Sentinel-2 images enables us to access a larger scale with freely available data and to access plant flowering timing with a ~5-day frequency of observations (Fig. 7). Furthermore, an advantage of this method is that it uses well-known techniques. First, the deep learning detection model—based on VGG16^35^, which is already a classical deep learning model in the field of computer vision—made the *Pleroma* detections for each Sentinel-2 images independently. Then, with the mean monthly frequencies of detections and the Fourier Tranform (FT), the continuous time series of flowering were used to find the points of interest for flowering phenology: days of start, peak and stop. This approach does not require direct analysis of the time series of Sentinel-2 images, just the detection time series, which simplifies the overall data processing. For the deep learning part of the method, one Sentinel-2 image is downloaded, pre-processed and the detection mask predicted in ~2 min. With the Atlantic Forest complete dataset, this process still required a consequent processing of more than two months, but it was feasible on a single computer with around 10 terabytes of storage that was equipped with a graphic card capable of running deep learning. While this seems like a lot of processing time, it consisted of detecting *Pleroma* trees in a huge amount of images, that is, 216,307,200 images. If one second was needed to do the detection for one image, it would translate into 6.86 years. Another advantage of the deep learning method is that no atmospheric correction of the images is needed before the detection step. While this can be counter intuitive for the remote sensing community, it is obvious from the computer vision perspective. In computer vision, a processing method called data augmentation is systematically used before sending images to the deep learning model. The objective of this step is to modify the characteristic of the image (e.g. hue, contrast, rotation angle,…) to increase artificially in the number of images seen by the model. This is made in order to increase the generalization power of the algorithm, that is, how perform the trained model on a new dataset^43^. In remotely sensed image, atmospheric condition and sunlight variations naturally create data augmentation (e.g. changes in illumination, contrast or brightness) that amplifies the model’s ability to generalize. During the training, only vertical and horizontal flips were used in the data augmentation step. The consistency of our results confirm that the detection model has a good generalization power and is able to detect objects of interest independently of atmospheric and illumination conditions.

The combination of human-level detection ability, machine consistency and computational power is what will likely make deep learning an essential tool for the remote sensing of vegetation^29,31^. Trees with certain characteristics that make them suitable for current deep learning algorithms, such as being abundant and easily identifiable in remote sensing images, can now be mapped on their entire distribution range. These distribution maps could be used support the urgent mapping of biodiversity from space^44–46^, for example, to avoid secondary forests dominated by *Pleroma* that can’t be detected otherwise, and to help identify ecological mechanisms that govern natural plant distribution and range, which remains one of the biggest questions in ecology^47^.

## Materials and methods

### Study site

The study covered the Brazilian Atlantic Forest domain (Fig. 2), which is located on the east coast of Brazil between latitudes 5° and 30° south, expanding over 500 km inland in the south. It consists of a total area of 1,085,151 km^2^ with limits defined by the Brazilian Ministry of the Environment^48^. The total area covered by Sentinel-2 tiles overlapping with the Atlantic Forest domain is ~2,006,959 km^2^. This latter area was used to compute the descriptive statistics of detections.

### Data

#### Sentinel 2 images

The pink or magenta blossoms of *Pleroma* trees were mapped using Sentinel-2 multi-spectral data with 10 m spatial resolution taken approximately every five days under the same viewing conditions. We used only Sentinel-2 images with Level-1C correction—which are orthoimage products, i.e. map projections of acquired images using a digital elevation model to correct ground geometric distortions—and delivered in images of 100 km × 100 km. Pixel radiometric measurements were provided in Top-Of-Atmosphere (TOA) reflectances (coded in 12 bits)^49^.

In the analysis, 213 Sentinel-2 tiles covering the Brazilian Atlantic Forest domain were used, totaling 2,006,959 km^2^ which is equivalent to ~20 billion Sentinel-2 pixels with 10 m spatial resolution (Fig. 2a). Amongst the 213 selected tiles, 36 had 2 orbits to download to obtain the full tile image due to the overlapping orbit paths (called replicates in the following text).

For each tile and replicate (213 + 36), the times series between 31 June 2016 and the 1 July 2020 was downloaded from the Google Cloud Storage Sentinel-2 repository (https://cloud.google.com/storage/docs/public-datasets/sentinel-2). To reduce the dataset size, we retained only images with less than 80% cloud cover; and, from the month outside the flowering months of the *Pleroma* trees (July to November), we kept only images with less than 25% cloud cover. The complete dataset was made up of 33,798 Sentinel-2 images.

Four spectral bands available at 10 m spatial resolution were used: Red (665 nm), Green (560 nm), Blue (490 nm) and NIR (842 nm). A border of 120 pixels with NA values was added to the image to produce images of 10240 × 10240 pixels to ease automation of the image analysis workflow, which generally works with 2^*n*^ × 2^*n*^ size pixel images. In our case here, the deep learning analysis was made with 128 × 128 pixel images and an additional 8 × 8 border. Sentinel L1C reflectance values are in the range of 0–10000 and were converted to 8 bits (0–254) with the following rules : for Red, Green and Blue bands, we kept the minimum value between 2540 and the original pixel value, divided this value by 10 and converted the result to integer; and for the NIR band, we keep the minimum value between 2540 and the original pixel value divided by a constant equaling 3.937, divided this value by 10 and converted the result to integer. While it was not expected to have RGB pixel values for vegetation with reflectance above 2540, it occured frequently for the NIR values. Dividing the NIR band values by the constant 3.937 enabled scaling the full range of the original NIR values between 0 and 2540 without losing too much information. For each tile, all 4 bands were saved in one GeoTIFF of 8 bits to ease storage and processing. The size of the complete dataset was 5.59 teraoctets. The automatic download, scaling and conversion of the images to 8 bits took about 25 days (from 16 July 2020 to 3 August 2020 and from 10 September 2020 to 13 September 2020).

#### Environmental data

To test the association of *Pleroma* trees with elevation and slope, elevation data from the Shuttle Radar Topography Mission (SRTM) were used^50^ (Fig. 12a). Specifically, we used the 3 arc-seconds (~90 m) spatial resolution digital elevation database (version 4) provided by the CGIAR Consortium for Spatial Information^51^. This dataset, in comparison to the original NASA STRM dataset, has been processed to fill data voids. From this dataset, we used the variables elevation (m) and computed slope (°) considering the four neighbor pixels (Fig. 12b). To analyse the relationship between *Pleroma* trees presence and forest tree cover, we used the tree cover percentage for the year 2000 at 30 m of spatial resolution, which we obtained from the global forest cover dataset (Fig. 12c), which is based on Landsat time series^52^.

The association of *Pleroma* trees with local climate was tested using the annual means of precipitation and air temperatures (Fig. 12d–f). The mean annual precipitation over the study period was computed from the CHIRPS v2p0 monthly precipitation dataset at 0.05° of spatial resolution produced by University of California, Santa Barbara (UCSB). CHIRPS data are global rainfall estimates from rain gauges and satellite observations^53^. The mean of maximum and minimum air surface temperatures over the study period were computed from the Aqua/AIRS L3 Daily Standard Physical Retrieval (AIRS-only) at 1° of spatial resolution V7.0 (AIRS3STD). AIRS, the Atmospheric Infrared Sounder on NASA’s Aqua satellite, gathers daily infrared energy emitted from Earth’s surface and atmosphere globally and provides 3D measurements of temperature and water vapor through the atmospheric column^54^. The annual mean of minimum and maximum air surface temperatures was calculated using the daily air surface temperature measured from the descending orbital pass, which occurs at 1:30 am local time (’SurfAirTemp_D’), and the ascending orbital pass, which occurs at 1:30 pm (’SurfAirTemp_A’).

Additionally, maps produced by the Brazilian Institute of Geography and Statistics (IBGE) of the geomorphological units and rivers of Brazil were used to describe the spatial distribution of the blossom detections^33^.

All environmental variables were resampled to a raster of 1280 × 1280 m spatial resolution using an average interpolation to match the resolution of the *Pleroma* tree detection dataset.

### Model

#### Neural network architecture

**Figure 13:**
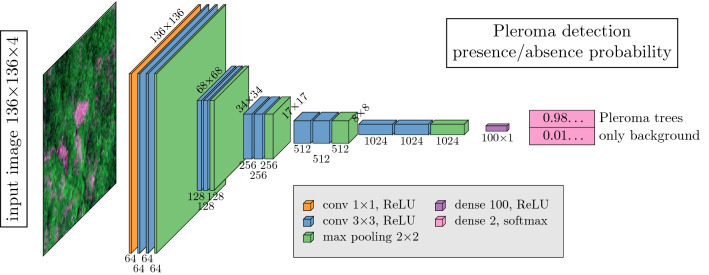
Architecture of the *Pleroma* blossom detection model.

This detection model is a deep learning neural network (Fig. 13), more specifically an encoder with a VGG16-like structure^35^, that given an image (input image) return the probability of presence of *Pleroma* trees with flowers in the image. The model inputs are 4 bands RGB-NIR images made up of 136 × 136 pixels at 10 m of spatial resolution (Fig. 13). Sentinel-2 tiles of 10240 × 10240 pixels were cropped based on a regular grid of 128 × 128 pixels, and 4 neighbouring pixels were added on each side to create an overlap between the patches. The resulting images are 136 × 136 pixels in size. However, in the training, the presence or absence of blooming *Pleroma* was given only for the images of 128 × 128 pixels without consideration of the borders. This enable to avoidance of the border effect that is common in convolutional neural networks. Each image of 136 × 136 pixels goes through a data augmentation process that consists in random vertical and horizontal flips. No additional data augmentation necessary due to the natural data augmentation provided by atmospheric conditions and illumination. After data augmentation, the images were then fed to the detection encoder. The encoder was made up of 5 consecutive convolution and pooling blocks, one fully connected layer (dense 100) and a final output layer with a softmax activation that provided the probability of presence of blooming *Pleroma* trees in the image (Fig. 13). Additionally, one drop-out layer was used at the end of the fully connected layer to perform further implicit data augmentation and avoid overfitting during training. The model has a total of 25,448,686 parameters, of which 25,440,622 are trainable. The model was coded in R language^55^ with Rstudio interface to Keras and TensorFlow 2.2^56–59^.

#### Network training

To make the training sample, a manual sample was produced for the Sentinel-2 tile 23KMQ, in the area where we had previously made a high resolution map of blooming *Pleroma*^6^, and for five other tiles where flowering *Pleroma* were detected visually from high resolution Google Earth images (22JFQ, 22JGQ, 23KLP, 23KLQ and 23KNQ, respectively). What is identified in the Sentinel-2 images are forest stands dominated by *Pleroma* and not single individuals. *Pleroma* trees have a small stature (8–12 m height) and crown of less than 10 m and one tree alone cannot influence sufficiently the reflectance to be clearly detectable in Sentinel-2 images. However, they occur very frequently clumped together, a common behaviour of this pioneer Genus. These flowering *Pleroma* dominated forest stands were easy to identify visually in the Sentinel-2 images because they combined several very distinctive features. First, an intense magenta-to-deep-purple color, which is an unusual color for other land covers in this ecological domain. Second, these identified *Pleroma* pixels should be undoubtedly identified as forested pixels and have a green color outside the blooming season. Third, *Pleroma* dominated forests often formed continuous magenta-to-deep-purple patches of size ranging from some 10 m × 10 m pixels to more than thousands of pixels and the shape of the patches tend to present linear features, likely representing the border of the space that was colonized by the *Pleroma* trees. Fourth, individual crowns were not visible, and the texture of the patches was very smooth during the blooming season with sometimes some inclusions of tree crowns of green color. Finally, texture of the *Pleroma* dominated forest stands outside the blooming season shown a smooth green texture, more homogeneous and with less shade than other forests. The first sample was constituted of images of background and of blooming *Pleroma* dominated forest stands that were following the previously described criteria. From this sample, we train a first model and applied it to the complete time series of Sentinel-2. From the results of this model, we obtained a first map of *Pleroma* trees and were also able to identify the main detection errors of this model, mainly clouds and dirt roads proximity with some unidentified agriculture fields or sometimes Eucalyptus plantation. The results of this first model were checked visually to produce a second larger sample (which was used for the results presented in this study) made up of images containing blooming *Pleroma* dominant to monospecific forest stands, a set of background images without blooming *Pleroma* and images identified erroneously by the first model as containing blooming *Pleroma*. While a large majority of the detected pixels were undoubtedly forest stands dominated by *Pleroma* trees, some other isolated trees of the genus *Handroanthus* (*Ipê* in Brazil or *Lapacho* in Argentina) with pink flowers and large crowns covering several pixels of 10 m × 10 m were also detected and kept in the training sample. For these particular *Handroanthus* trees, crowns were visible during and sometimes also outside the blooming season, which was not the case for detected *Pleroma* dominated forest stands. Finally, as our model detected also large Handroanthus trees, we must acknowledge that other tree species with highly similar features could also potentially being detected.

The final training samples comprised a total of 158,612 images of 136 × 136 pixels. Among them, 35,541 contained blooming *Pleroma* trees and 123,071 images contained only background. Among the background images, there was nine different images types: images without blooming *Pleroma*, i.e., background such as other land covers, urban structures, water surfaces and agriculture and other land uses (57,007), images with forests containing *Pleroma* but outside the flowering period (23,427), images with clearly identified detection errors mainly located in the east of the São Paulo state (12,965), clouds and detection errors in clouds (10,991), images clearly identified as detection errors near the State of Bahia (9030), other detection errors over Atlantic Forest (5843), images of forests without *Pleroma* trees during the season of blooming (2170), images with identified detection errors in the northern part of Atlantic Forest (1126) and images with no data (512). Of these images, 80% (126,890) were used for training and 20% (31,722) used for validation.

During network training, we used a standard stochastic gradient descent optimization, a binary cross-entropy loss and the optimizer RMSprop^60^ with a learning rate of 1e-4. We used the accuracy (i.e. the frequency with which the prediction matches the observed value) as the metrics of the model. However, due to the imbalance between the number of blooming *Pleroma* and background images, the metric of the model was weighted by one for the background and, for the *Pleroma*, by the ratio between the number of background images and the number of images containing blooming *Pleroma*: that is, ~3.5. The network was trained for 5000 epochs, where each epoch was made of 61 batches with 2048 images per batch and the model with the best weighted accuracy was kept for prediction (epoch 4331 and weighted accuracy of 99.58%). The training of the models took approximately 9 hours using a Nvidia RTX2080 Graphics Processing Unit (GPU) with an 8 GB memory.

#### Prediction

To avoid border effects, each 10240 × 10240 pixels Sentinel-2 image was cropped on a regular grid of 128 × 128 pixels (1280 × 1280 m), and 4 neighboring pixels were added on each side to create an overlap between the patches. The function gdal_retile^61^ was used for this operation. Prediction was then made for each subset image: for each image, the detection model returned 0 or 1 if a blooming *Pleroma* was found in the image. Then the results were spatialized again using the grid, but this time, each cell of the grid only received 1 value, the prediction, resulting in a raster of 80 columns and 80 rows and a spatial resolution of 1280 m, of the same extent as the Sentinel-2 image. The value of the pixels (1 or 0) indicated the presence or absence of blooming *Pleroma* trees in this squared area of 1280 m of side. Prediction using GPU of a single tile of 10240 × 10240 pixels took approximately 1 minute on a Nvidia GTX1080 with an 8 GB memory and 45 s on a Nvidia RTX2080 with an 8 GB memory. The prediction for the complete Sentinel-2 time series presented in this work took approximately 22 days using a Nvidia GTX1080 GPU—from the 30 October 2020 to the 20 November 2020.

### Spatio-temporal analysis

To analyse the seasonality of the detections, daily maps of flowering *Pleroma* detections were produced for the studied period on a grid overlapping the entire Atlantic Forest (projection UTM 23S and datum WGS84) with a spatial resolution of 1280 m to match the resolution of the predictions. For each day, each pixel of the grid was given a classification: observed with flowering *Pleroma*, observed without flowering *Pleroma*, observed with clouds (using the cloud cover mask for Sentinel-2 images of this day) or as non-observed (no image or NA data for the pixel on that day). These daily grids were use to produce the map of flowering *Pleroma* trees (number of detections of flowering *Pleroma* for each pixel along the time series), the map of the total number of observations per pixel and the map of the total number of observations without clouds.

To analyse the seasonality of blooming, the detection results were aggregated by month—even with a 5-day frequency there were still too few observations to analyse each annual timing and duration of flowering, and changes of the flowering dates between years were not expected based on the existing botanical information of the species. For each pixel, the number of detections per month were divided by the total number of observations without clouds per month. This enabled to normalized the detection values between zero and one and made sense given that we were not interest in the number of detections but rather in the times of the year when the number of detections was the highest: the peak of the blooming.

To find the characteristics of these time series—one or more blooming peaks and the days when the blooming begins, peaks and stops—the normalized time series of mean monthly observations of flowering *Pleroma* were filtered using the Fourier transform (FT) (Eq. 1). This decomposition was made the keeping only the annual, bis- and tris-annual frequencies that compose the blooming signal, and to provide a continuous representation of the discrete blooming observations. In other worlds, the Fourier transform of the normalized time series observations enabled to model and compute the values of blooming for each day of the year and better estimate the days blooming started, peaked, and ended. While initially, a decomposition with only annual and biannual frequencies was expected to fit well to the times series (as more than two peaks per year were not expected), it appeared that when the two peaks were close in time (such as in a 2–3 month interval), only annual and biannual frequencies were not sufficient to give a good model of the signal, and the triannual frequency was added to resolve this issue. Furthermore, it was assumed that other periods in the signal were only constituted by noise.

The blooming signal was modelled by the following equation:

1 $$\begin{aligned} {\widehat{bloom}}(t)& = bloom_0 + pow_0 \,\left( p_{4} \sin \left( 2\pi \frac{1}{4} t + \rho _4\right)  \right. \\ & \quad\left. + p_{6} \sin \left( 2\pi \frac{1}{6} t + \rho _6\right) + p_{12} \sin \left( 2\pi \frac{1}{12} t + \rho _{12}\right) \right) \end{aligned}$$

with $p_4 + p_6 + p_{12}=1$ and for $t=1,\ldots ,12 \times n$, ${\widehat{bloom}}$ is the filtered blooming time series; $bloom_0$ is as an estimate of the mean annual blooming; t is the time in months; $\rho _4$, $\rho _6$ and $\rho _{12}$ are the delay of signal components with periods of 4 months, 6 months and 12 months, respectively; $pow_0$ is the power of the signal and $p_4$, $p_6$ and $p_{12}$ are the relative proportions in the signal of the periods of 4 months, 6 months and 12 months, respectively.

To ease optimisation and cohere with the biological significance of our model, some data cleaning and adjustments were made. First, pixels with less than 4 observations over the 4-year period were removed from the analysis. Second, isolated peaks with only 1 or 2 observations during the 4-year period and between months without *Pleroma* detection were set to 0. Third, all the values of the normalized blooming time series were multiplied by 10, which seemed to ease the convergence of the optimisation algorithm. Fourth, the first months before and after the blooming period were set to a negative value equal to − 0.15 × the maximum value of the pixel time series. This was made based on the assumption that blooming is quite fast (based on the observation data) and happens between the month when the blooming is first observed and the previous month (and when blooming is last observed and the next month), and it forced the model to go below the 0 value during this period. Fifth, a weight was added to each point corresponding to its value, as we were interested in estimating accurately the peak value. A weight value of 0 was set to the month with a 0 value, and a weight of 1 was set to the months with negative blooming values (pre- and post-peak months). Finally, to facilitate the optimization, the time series of values and weights was replicated 3 times (*n* = 3). While this did not change the periodicity of the signal, it enabled to estimates better the value of the first and last month of the time series, as well as to ease optimisation. The parameters $bloom_0$, $pow_0$, $p_4$, $p_6$, $p_{12}$, $\rho _4$, $\rho _6$ and $\rho _{12}$ were then estimated by a weighted least square minimization using the weights previously described. The accuracy of the model was given by the weighted R^2^ computed with the observed and predicted values of blooming for each month. As the Fourier transform is highly flexible, it can adjust almost perfectly to the data: the median of weighted R^2^ was close to 1 (with a 95% confidence interval—from percentile 2.75 to 97.5—of 0.86.0 to 1).

**Figure 14:**
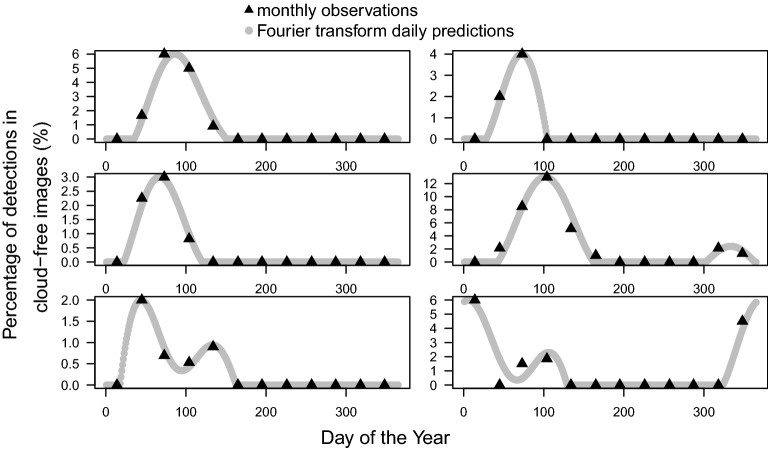
Examples of observed time series of detections in cloud-free images (%) and their daily estimation modeled using the Fourier transform.

After the decomposition of the blooming signal, a daily time series of 1 year of ${\widehat{bloom}}$ was computed with the obtained parameters (365 values) (Fig. 14). Daily values of months with a weight of 0 were set to 0 as well as predicted negative values. Then all peaks and pits were identified in the ${\widehat{bloom}}$ time series. A peak or pit is an observation that is preceded and followed by, respectively, lower or higher observations^62,63^. For each peak, the day of start and stop were identified using the pit values. After this analysis, we were able to describe the blooming time series: that is, if there were one or more peaks and, for each peak, the days when the blooming initiated, peaked and stopped.

To determine if different populations could be identified based on flowering timing, a cluster analysis was performed. A classical K-means clustering analysis was made on a dataset containing, for each pixel where *Pleroma* were detected, the days of start, peak, and end of blooming, the associated normalized blooming values and the *xy* coordinates of the pixels. If there were two peaks for a pixels, a line for each peak was created in the dataset. As the *xy* coordinates were in metres, they carried most of the variance in the dataset. To avoid the artefact of having clusters based only on the distance between pixels, the *xy* coordinates were divided by 100,000 and rounded to the nearest unit. Before the clustering analysis, all variable were scaled and centered. The number of clusters was determined based on the curve representing the total within-cluster sum of squares as a function of the number of clusters, and also to have the maximum number of clusters.

To describe the association of *Pleroma* trees with environmental variables, we first reclassified each environmental variables into 10 classes according to the variable’s quantiles. Then a bootstrap procedure was applied. For the number *N* of *Pleroma* trees detections, *N* random point locations were sampled within the Atlantic Forest domain, and the value of each environmental variable at each point was extracted and stored. This operation was repeated 100 times. It enabled to compare the number of *Pleroma* trees in each quantile class with the mean and gave us a 95% confidence interval for the number of points obtained by random spatial sampling in each class. Using the elevation as an example, the null hypothesis of no spatial association between *Pleroma* trees and elevation was rejected at a level of 0.05% if the number of *Pleroma* trees in a quantile class of the elevation was outside the (0.025, 0.975) quantiles of the empirical distribution of elevation obtained by random location sampling in the same class. The same analysis of association with the environmental variables was made for the *Handroanthus* population identified by the K-means clustering analysis.

All analyses were performed using R project software^55^.

## Supplementary Information

Supplementary Information 1.

Supplementary Video 1.

## Data Availability

The data that support the findings of this study are all publicly available from their sources. Processed data, products and codes produced in this study are publicly available on Zenodo https://doi.org/10.5281/zenodo.4549662 or from the corresponding author upon request.
