# Supplementary material for: The flowering of Atlantic Forest Pleroma trees
Source: Sci Rep. 2021 Oct 14;11:20437. doi: 10.1038/s41598-021-99304-x (PMC8517022; doi:10.1038/s41598-021-99304-x)
Supplement: Supplementary file 1 — Supplementary Information 1. [file 41598_2021_99304_MOESM1_ESM.docx]

**Detections.mp4**: Video of the daily blossoms of pink and magenta colours in the Atlantic Forest domain estimated from the detections of the deep learning model trained with images of blooming *Pleroma* trees. The colour of the pixel indicates the blooming peak date.
